# RNA-Seq of Single Fish Cells – Seeking Out the Leukocytes Mediating Immunity in Teleost Fishes

**DOI:** 10.3389/fimmu.2022.798712

**Published:** 2022-01-24

**Authors:** Justin T. H. Chan, Safwen Kadri, Bernd Köllner, Alexander Rebl, Tomáš Korytář

**Affiliations:** ^1^ Institute of Parasitology, Biology Centre of the Czech Academy of Sciences, České Budějovice, Czechia; ^2^ Helmholtz Zentrum München, Institute of Lung Biology and Disease, Regenerative Biology and Medicine, Member of the German Center for Lung Research (DZL), Munich, Germany; ^3^ Institute of Immunology, Friedrich Loeffler Institute, Federal Research Institute for Animal Health, Greifswald, Germany; ^4^ Institute of Genome Biology, Research Institute for Farm Animal Biology, Dummerstorf, Germany; ^5^ Faculty of Fisheries and Protection of Waters, University of South Bohemia, České Budějovice, Czechia

**Keywords:** RNA-seq, flow cytometry, antibody, leukocytes, lymphocytes, phenotype, single-cell transcriptome, teleost bony fish

## Abstract

The immune system is a complex and sophisticated biological system, spanning multiple levels of complexity, from the molecular level to that of tissue. Our current understanding of its function and complexity, of the heterogeneity of leukocytes, is a result of decades of concentrated efforts to delineate cellular markers using conventional methods of antibody screening and antigen identification. In mammalian models, this led to in-depth understanding of individual leukocyte subsets, their phenotypes, and their roles in health and disease. The field was further propelled forward by the development of single-cell (sc) RNA-seq technologies, offering an even broader and more integrated view of how cells work together to generate a particular response. Consequently, the adoption of scRNA-seq revealed the unexpected plasticity and heterogeneity of leukocyte populations and shifted several long-standing paradigms of immunology. This review article highlights the unprecedented opportunities offered by scRNA-seq technology to unveil the individual contributions of leukocyte subsets and their crosstalk in generating the overall immune responses in bony fishes. Single-cell transcriptomics allow identifying unseen relationships, and formulating novel hypotheses tailored for teleost species, without the need to rely on the limited number of fish-specific antibodies and pre-selected markers. Several recent studies on single-cell transcriptomes of fish have already identified previously unnoticed expression signatures and provided astonishing insights into the diversity of teleost leukocytes and the evolution of vertebrate immunity. Without a doubt, scRNA-seq in tandem with bioinformatics tools and state-of-the-art methods, will facilitate studying the teleost immune system by not only defining key markers, but also teaching us about lymphoid tissue organization, development/differentiation, cell-cell interactions, antigen receptor repertoires, states of health and disease, all across time and space in fishes. These advances will invite more researchers to develop the tools necessary to explore the immunology of fishes, which remain non-conventional animal models from which we have much to learn.

## Introduction

In the year 1891, while admirers of classical music basked in Tchaikovsky’s Nutcracker, while Maria Skłodowská (married name “Curie”) entered Sorbonne University, and while Victorinox started production of its famous Swiss army knife, Joseph Lister, the respected founder of aseptic technique in surgery, brought together the leading proponents of the conflicting concepts of cellular and humoral immunity, namely Élie Metchnikoff and Paul Ehrlich, respectively, for the *Seventh International Congress of Hygiene and Demography* held in London, England. The controversial discussions between both schools became a highlight of scientific conferences and remained heated for years. Although both schools started to realize the complementarity of their, at the time, opposing views, it was Almroth Wright’s experiments showing cooperation of antibodies and phagocytes in bacterial engulfment and killing that led to amalgamation of both concepts. In 1908, both Metchnikoff and Ehrlich, were awarded the Nobel Prize in Physiology or Medicine and sowed the seeds of modern immunology: the concepts of complementarity between innate and adaptive immunity, between humoral and cell-mediated immunity (1).

In the 100 years that followed, driven by researchers’ curiosity and technological advances, the field grew, leading to seminal discoveries and paradigm shifts revealing the immense complexity of the immune system and its main players. The field was further propelled forward by hybridoma technology and progress in molecular biology. However, it is not purely technological advances that have driven progress, but also novel perspectives, points of view and comparisons. Nowadays, in traditional mammalian models, we have a comprehensive understanding of the individual leukocyte subsets, their phenotypes and their specific roles in health and pathology, allowing targeted strategies for improving our health and combating diseases. Nevertheless, while the mammalian-centric perspective provided invaluable insights into the diversity of leukocytes and deciphered the underlying mechanisms of their interactions resulting in protective immunity, it leaves us wondering about the forces that have driven evolution and diversification of immunity to the present day. Looking back, it is thanks to non-traditional models as much as it is to traditional (mammalian) models that we now understand leukocyte function. Metchnikoff himself based his theories on starfish larvae (1). The fascination of Jules A. Hoffmann with fruit flies resulted in the discovery of Toll genes and their role in recognizing pathogens (2). We often forget that the “B” in B cells stands for the Bursa of Fabricius in chickens, from which Max D. Cooper et al. so elegantly dissected the developmental origins of the two separate lymphocyte lineages responsible for humoral and cell-mediated immunity (3). More recently, research on non-mammalian models provided crucial insights into the phagocytic capacity of B cells and thrombocytes (4, 5), into the evolution of mucosal immunity (6), or into the ability to ‘smell’ viruses (7) and exploit niches with high temperatures for faster healing (8) – all of which have changed the paradigms of mammalian immunology.

All this to say that non-conventional animal models have as much left to teach or reveal to us as mammalian models had in the past and still have today. Evolutionarily successful (convergent) mechanisms such as somatic diversification of antigen receptors are universally conserved among all extant vertebrates, proving that they are essential for survival of the species; whereas mechanisms more pronounced in, or exclusive (divergent) to ancestral clades offer us innovative mechanisms and solutions for the same problems all complex organisms face: how to coexist with microbial life that is unimaginably diverse, everchanging, and omnipresent.

## Fish as a Model for Evolutionary Immunology

Fishes are the oldest phylogenetic class of animals protected by both innate and adaptive immunity, as we know them from mammalian models (9). At the cellular level, they are equipped with all major populations of leukocytes collaborating synergistically on innate and adaptive immune responses. Nevertheless, although basic elements correspond in fish and mammals, many distinct characteristics and unique cellular features make up the composition and function of the fish immune system (10). In the absence of lymph nodes, the main lymphoid organs are the spleen and head kidney, which provide niches and meeting points for the classical trio of antigen-presenting cells (APCs), B cells and T cells, supporting the proliferation and differentiation of cognate lymphocytes (11, 12). As in other vertebrates, myeloid lineage leukocytes are activated by pathogens. They take up antigen, break it down into peptides, and present it to T cells *via* major histocompatibility complexes (MHCs). Then, CD4^+^ T cells orchestrate responses by secreting cytokines that provide mitogenic, differentiation, and/or polarization signals (13). Teleost cell-mediated immunity generally resembles that in higher vertebrates and is mediated by natural killer (NK)-like cells and CD8^+^ T cells whereas antibody-mediated humoral defense is provided by B cells, which secrete three classes of antibodies (IgM, IgD and IgT/Z) (6, 14–17).

**Figure 1:**
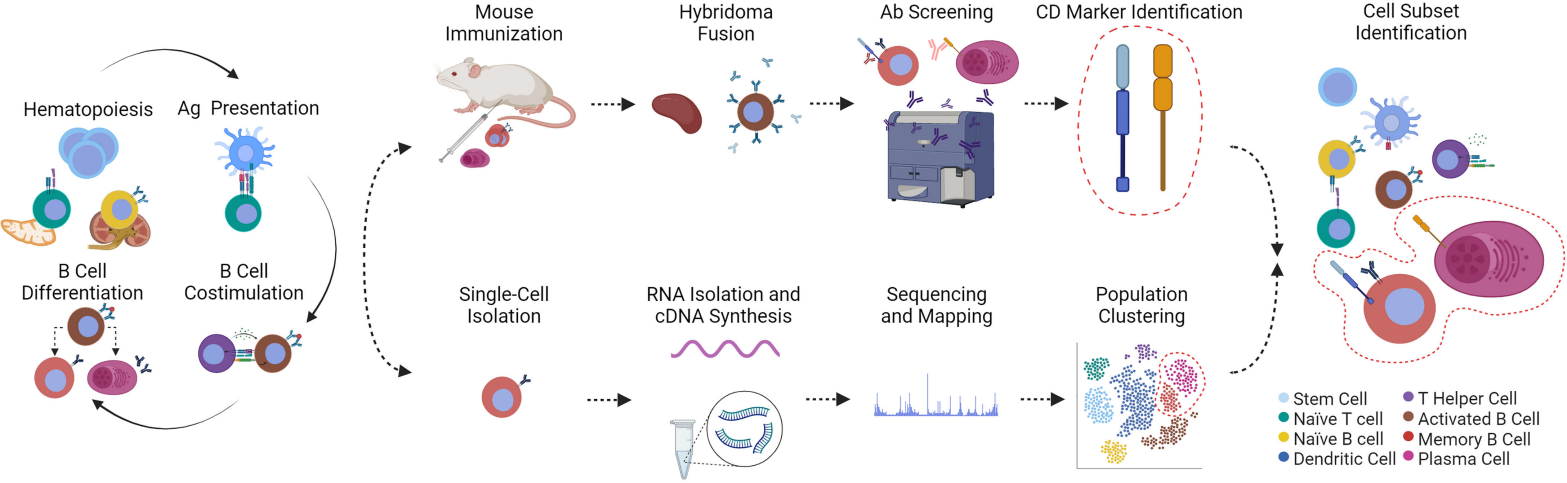
(Immuno)phenotyping and discovery of fish leukocyte subsets *via* hybridoma technology or scRNA-seq technology are presented in chronological order from left to right. (Hematopoiesis) All leukocytes arise from hematopoietic stem cells in the thymus or kidney of fishes. (Ag Presentation) Following activation and co-stimulation by antigen (Ag)-presenting cells, these mature lymphocytes are themselves precursors for differentiated T helper cells that activate B cells (B Cell Costimulation). (B Cell Differentiation) B cells then follow at least two fates into memory B or plasma cells. Immune responses therefore generate a plethora of immune cell subsets. The cells involved can be identified by hybridoma technology or scRNA-seq. (Top row, from left to right) As pioneered by Köhler et al. (18), unidentified immune cell subsets can be used as immunogen for laboratory animal immunizations; antibody-secreting cells from splenic tissue can be fused to immortalized myeloma cells. The resulting “hybridomas” produce monoclonal antibodies that are screened for reactivity to fish leukocytes. Reactive antibodies are used to identify the bound cluster of differentiation marker (CD marker) (19) that are then associated with a specific leukocyte population. (Bottom row, from left to right) Alternatively, thousands of single cells are isolated, from which total RNA is reverse-transcribed, the complementary DNA sequenced and mapped to transcriptomes for comprehensive clustering of a heterogeneous population of cells based on hundreds of shared transcripts. To achieve the same result with traditional hybridoma technology would require isolation of hundreds of monoclonal antibodies and their corresponding (surface) markers. The cytoplasm of cells presented in this figure are color-coded based on the legend on the bottom right. The phenotyping and identification of memory B and plasma cells is shown as an example. Created with BioRender.com.

Progress in fish immunology is closely tied to a revolutionary approach in immunology: hybridoma technology published by Georges Köhler and César Milstein in 1975, which enables, in immortalized cell lines, the unlimited production of a monoclonal antibody (mAb) with specificity to a single distinct antigenic epitope (**Figure 1**) (18). Their Nobel Prize-winning method was applied shortly after towards isolating the first MHC-specific mAbs (20). Since then, hundreds of thousands of mAbs were isolated allowing the identification of surface markers on leukocyte populations throughout development, differentiation, activation, proliferation and effector function processes. As early as 1982, the rapidly increasing number of mAbs specific for leukocyte surface markers (mainly on human and mouse leukocytes) required a universally accepted nomenclature named “Clusters of Differentiation” (CD) (19). To date, over 350 CD molecules and their corresponding mAbs with a similar reaction pattern have been defined in 11 Human Leukocyte Differentiation Antigen Workshops. Discovery of human and mouse CD molecules has been closely followed by establishment of similar panels of markers in other species (rat, rabbits, and farm animals) in the past two decades.

Fish immunologists recognized the immense potential of the new technology and by 1979, only four years after Köhler’s publication, the first mAbs specific for fish leukocyte markers were published by Greg Warr (anti-trout IgM) (21), followed by the development of anti-carp thymocyte mAbs by Chris Secombes in 1983 (22). Using the classical approach of immunization with purified immunoglobulin or a mixture of isolated thymocytes, they identified 27 hybridoma clones specific for carp thymocytes and 18 clones targeting serum immunoglobulin (Ig) (22). Encouraged by these results, a number of groups worldwide established similar CD-like panels for different fish species (common carp, crucian carp, rainbow trout, salmon, seabass, and seabream) in the following two decades (23–25). Nevertheless, although classical immunization procedures helped isolate several antibodies targeting individual leukocyte subsets, immunologists also encountered obstacles on the path to establishing panels of CDs as in mammals. Since the first attempts, due to very limited inter-species cross-reactivity, researchers realized that the direct comparison and application of the established mAbs was not possible due to the evolutionary diversity of fish species. Additionally, in contrast to mammalian leukocyte CD markers, most of the surface markers defining teleost lineages could only be detected by mAbs but not identified. Instead, advances in molecular biology helped identify lineage-specific markers (such as *CD4*, and *CD8*) and producing these markers in prokaryotic and eukaryotic systems renewed research efforts. Nevertheless, due to the possible alternative codon usage and protein misfolding, the molecular methods facilitated production of only a handful of corresponding novel antibodies (26). Consequently, the production of mAbs has only proven successful against a few conserved leukocyte markers and populations, including against B cells, CD8^+^ and CD4^+^ T cells, monocytes, granulocytes, or thrombocytes, and only for a limited number of fish species (**Table 1**) (6, 15, 22, 25, 27–42). However, although these antibodies provided pivotal advances in our knowledge, they are not enough to dissect and study the remarkable heterogeneity within the investigated populations leaving many pressing questions about these cells unanswered: what are their precursors, their activation and differentiation profiles, their associated signaling molecules, and their lineage-specific transcription factors?

**Table 1:** Summary of available antibodies specific to rainbow trout *Oncorhynchus mykiss* or common carp *Cyprinus carpio* leukocytes.

	*TROUT*	*CARP*
*B cells*	IgM, IgD, IgT, MHC II, CD5 (6, 27–30)	IgM (22)
*T cells*	CD4-1, CD4-2, CD5, CD8α, CD3ϵ (15, 28, 31, 32)	CD4-1, CD8α, **WCL9**, **WCL38** (33–35)
*Dendritic cells*	Langerin/CD207 (36)	^-^
*Platelets*	**mAb 42 and others** (25)	**WCL6** (37)
*Natural Killer Cells*	-	-
*Macrophage*	CD4-1, **Mm**, **MGm**, **MGcp1**, **MGcp2**, **mAb 45**, **mAb 21** (31, 38–40)	**WCL15** (41)
*Monocytes*		**TCL-BE8** ^lo^ (42) **WCL15** (41)
*Neutrophils*	**mAb Q4E**, **Mm**, **MGm**, **MGcp1**, **MGcp2** (38) **mAb 21 (** 40)	**TCL-BE8** ^hi^ (42)
*Basophils*		-
*Eosinophils*		-
*Red Blood cells*	-	-

Either the protein marker bound is listed or (in bold text) the name of the monoclonal antibody is presented. The scarcity of markers and incomplete characterization of monoclonal antibodies (mAbs and the WCL series of mAbs, notably) means that certain lineages are tentatively merged, cannot be distinguished by available reagents, have no antibodies specific to them (-), or are detected by antibodies whose ligand has not been identified yet (in bold text).

The advantage provided by anti-CD marker mAbs has been documented using pan-sea bass T cell-specific antibodies (clone DLT-15) described in 1997 for the functional characterization of T cells, and analysis of the expression pattern of their T cell receptors (TCRs), CD4 and CD8 co-receptors as well as T cell-derived cytokines (24). The establishment of pan-T cell-specific as well as CD4- and CD8-specific mAbs allowed similar investigations in trout, ginbuna crucian carp, zebrafish, and other cyprinids including the common carp (15, 31, 33). Nevertheless, although the molecules forming the TCR complex, TCRαβ, and TCRγδ as well as CD3 have been known for two decades, few mAbs recognizing CD3ϵ have been established (32, 43, 44). Similarly, among lineage-specific markers (such as CD1, CD2, CD5, CD6 and CD7), only anti-rainbow trout CD5 has been isolated (28). Thus, for example, no tools are available to definitively demonstrate the existence of teleost counterparts to mammalian T helper subpopulations despite many studies having identified fish orthologues of cytokines involved in Th1, Th2, Th17 and Treg responses (45, 46).

Similarly, studies focusing on B cell biology and function in several species (e.g., common carp, crucian carp, trout, salmon, seabass) rely solely on antibodies specific for the antigen receptor IgM, IgT/IgZ, and IgD or their Ig light chain. Although these anti-immunoglobulin-mAbs (anti-IgM, anti-IgT, and anti-IgD) (6, 21, 47) facilitated the functional characterization of teleost B cells (48, 49), their involvement in mucosal immunity (6) and development (11), no mAbs are available to track the expression of lineage markers equivalent to mammalian CD19, CD20, CD79a/b. We face similar obstacles when analyzing the main B cell effector functions. Whether in infection models or challenge with model antigens, secretion of antigen-specific antibodies indicate that the B cells differentiate into antibody-secreting cells (50–54). However, with little information about the surface markers they express and their phenotypes, apart from the production of antibodies, we can only indirectly study this population. Similarly, based on their phagocytic activity, fish B cells were considered a subset of “natural” B cells (termed B1) (55). However, only a portion of all fish B1 cells is phagocytic, with no known marker that can tell apart B cells with and without this capacity. Finally, the presence of long-lived memory B and T cell populations is still under debate (56). Currently, teleost memory lymphocytes can only be defined by functional characteristics rather than specific markers: they are the B and T cells that have differentiated during an immune response, persisting long after resolution of this response, and awaiting reactivation.

Knowledge about teleost innate leukocytes is even more held back by the lack of tools. Innate immune mechanisms play a larger role in providing resistance to pathogens in poikilothermic fish. This is ensured by a greater variety and specialization of functional subpopulations of myeloid cells (monocytes, macrophages, granulocytes, dendritic cells) as characterized by distinct expression of surface markers for antigen recognition, antigen presentation as well as for cytokine receptors. Although mAbs recognizing myeloid cells in salmonids or cyprinid species are described, the corresponding surface molecules are not characterized yet and so the identity of these leukocytes is often imperfectly determined only by the morphological features in flow cytometry (42, 57–59). Despite phenotypic and functional differences, these cells are often referred to as myeloid cells. Similarly, thrombocyte subsets that are nucleated in evolutionarily old teleosts, need to be further characterized for antigen recognition and presentation activities. Although, a panel of mAbs recognizing this leukocyte population was used to investigate thrombocyte biology in trout and carp, this has been applied without any basic information about the antigens targeted and their functions (23, 60). As for dendritic cells, they have been indirectly identified in zebrafish by their ability to phagocytose labeled bacteria and by their glycosylation profile *via* binding to peanut agglutinin, an alternate (lectin) affinity reagent that is specific to a disaccharide rather than to a particular protein (61).

**Figure 2:**
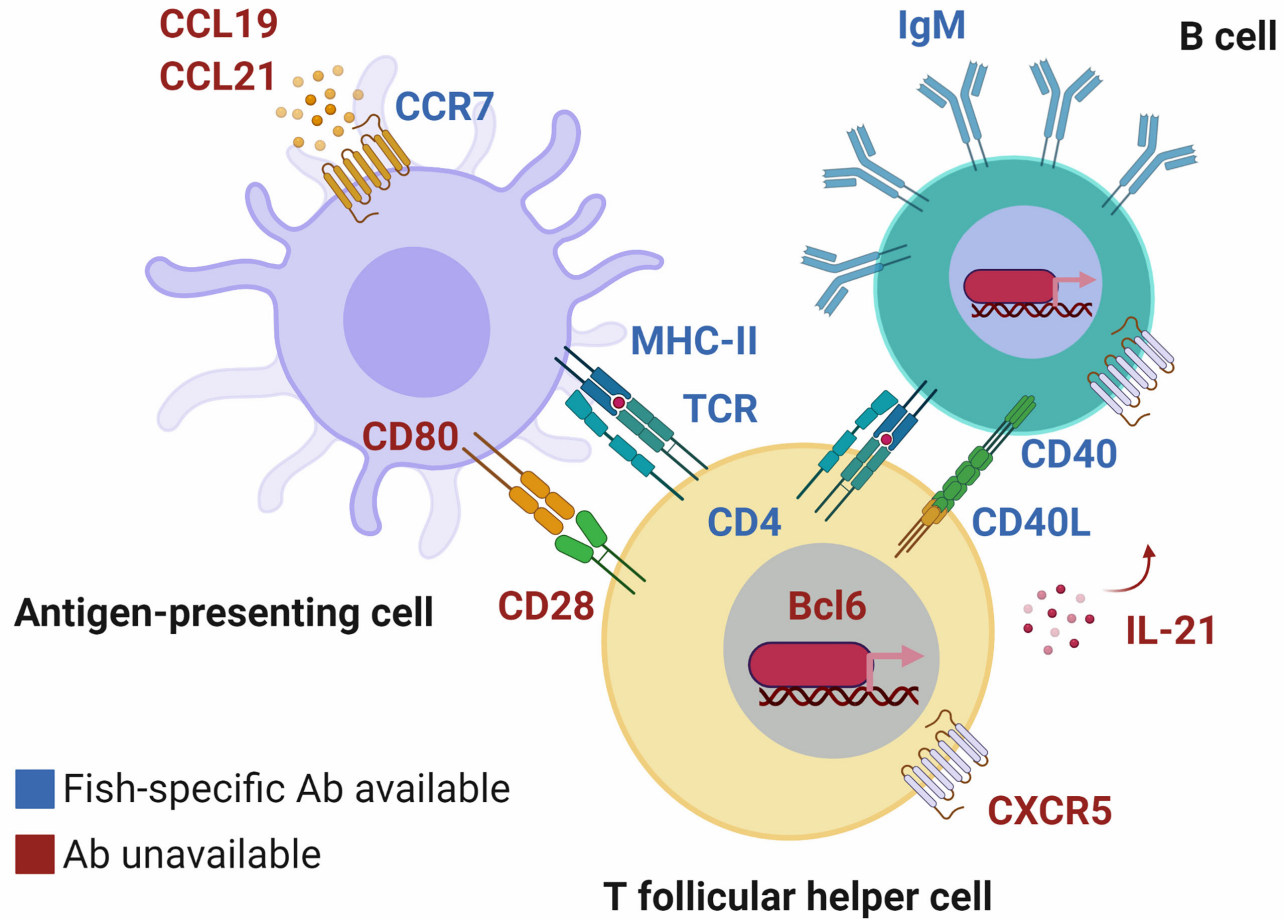
T cells, B cells and dendritic cells interact *via* surface molecules to mount an adaptive immune response. These molecular interactions inform us about the “where”, “when”, and “how” the process takes place. In fish, although orthologous markers have been identified (labeled in red text), only a select few have corresponding fish-specific antibodies that facilitate their study (in blue text) and they are not available for all species. Until we fill in the gaps, we will not know to what extent immune responses are mounted similarly or differently in mechanism, compared to mammals. Chemokine receptors such as CCR7 and CXCR5 ensure that pairs of antigen-presenting cells (APCs) and lymphocytes (and pairs of peptide-loaded major histocompatibility complex molecules [MHC]-T cell receptors [TCRs]) will encounter each other in specialized lymphoid niches. Co-stimulation *via* CD80/86 on APCs binding to CD28 on the surface of T cells, or CD40L-CD40 T cell-B cell interactions (62) lead to activation and differentiation *via* transcriptional regulators such as Bcl6. There is indirect evidence that immune responses are mounted in much the same way in fish, e.g., CD80/86 being capable of inducing cytokine production (63). However, examples such as *CCR7* being predominantly expressed by B cells in trout (discovered *via* an anti-trout CCR7 antibody) (64) highlight the need for additional tools, and phenotyping to determine discrepancies between evolutionarily distant vertebrates. Created with BioRender.com.

In comparison to what is available in mammalian models, the lack of targets and tools at the disposal of fish immunologists is holding the field back from describing even the basics of how APCs collaborate with T and B cells to mount an immune response. The phenotypic markers they express throughout the process help paint a complete picture of the trafficking/migration of leukocytes, differentiation, and multifaceted functions of lymphocytes (**Figure 2**). Upon activation and antigen uptake, APCs express the chemokine receptor CCR7, which binds to the chemokines CCL19 and CCL21 produced in lymphoid tissues. This receptor, along with CD80 and CD86, facilitate encounter in lymphoid tissues and co-stimulation respectively, between APCs and lymphocytes (65, 66). The nature of the antigen/peptide presented to a cognate T cell and environmental (cytokine) cues dictate its fate. Generally, activated T cells can differentiate into T follicular helper (Tfh) cells by responding to cytokines such as IL-21 and expressing the chemokine receptor CXCR5, *via* the transcriptional regulator Bcl6 (67). In turn, CXCR5 facilitates encounter of the Tfh cell with activated B cells presenting the same antigen/peptide. Paracrine/autocrine IL-21, along with molecules such as CD40 ligand, induce B cells into initiating the germinal center (GC) reaction (68, 69). The GC reaction is T cell-dependent and generates memory B and plasma cells as well as their antigen-specific affinity-matured antibodies. In summary, the complementarity of receptor-ligand pairs, cells and their niches paint a complete picture of the “where”, “when”, and “how” an immune response is mounted. Especially considering that the example presented here is simplified, that the process is circular in reality (e.g., signaling events are reciprocal), and that fish-specific antibodies are only available for select few markers and select fish species (**Table 1** and **Figure 2**, markers colored in blue) (6, 15, 22, 25, 27–42, 62–64), it is therefore difficult at the moment to detail the very same processes in fishes.

Additionally, even in mammalian species, the definition of leukocyte subsets is a constant work-in-progress due to complexity, locality, and species specificity. For instance, two markers such as CD19 and CD138 can define at least four subpopulations of human plasma cells in the bone marrow (70); the marker CD27 is exclusive to human memory B cells in circulation but in mucosal lymphoid tissues, such as the human tonsil, it is also expressed by activated T cells (71); CD27 is not used at all as a marker of murine memory B cells given that a majority of B cells lose it upon differentiation. By identifying orthologous or analogous markers in fishes, we could learn about the rich diversity of teleost leukocyte populations mediating these universally protective processes. Researchers have identified fish orthologues of a majority of these markers at the molecular level (63, 64, 72–77). These markers were focused on, sought and identified based on the assumption that they are relevant to fish immune processes equivalent to those in mammals. Although this appears to be the case for *GATA-3* whose expression corresponds with that of *IL-4/13A* in salmonids (77), the marker CCR7 was unexpectedly predominantly expressed by an IgD^+^ B cell subset in trout gills that help activated lymphocytes home to the head kidney (64). Given that even mice and humans do not always correspond, we need more context on how fish markers function, especially if their expression or roles in fishes diverges from those in their mammalian counterparts.

In a way, whether it is by size or granularity, or whether it is by activities such as phagocytosis or antibody secretion, we are still partly dependent on identifying leukocyte subsets by their morphologies and activities much like Metchnikoff did under the microscope. Given what little we have to work with (**Table 1**) and how little we know, fish immunologists stand to gain the most from alternate methods for cell phenotyping. Therefore, how do we phenotype fish leukocytes comprehensively, in a way that takes their complexity and unknowns into consideration?

## Following the Lead

Our current understanding of the function, complexity and heterogeneity of mammalian leukocytes is a result of decades of concentrated efforts, aiming to identify markers and populations using conventional methods of antibody screening and antigen identification (**Figure 1**). However, in light of current advancements in sequencing technologies, methods of the past have become too laborious, ineffective, inefficient, and costly long-term. Having reached a limit of what could be achieved with existing antibodies, fish immunologists sought to overcome it through innovation. One such model in which such efforts have been rewarded is in zebrafish (*Danio rerio*) using antibody-independent microscopy studies of leukocyte populations. The transparency of zebrafish embryos and larvae combined with their amenability to genetic manipulation allowed for *in-vivo* imaging of transgenic hematopoietic cells (78, 79). Viable transgenic leukocytes are one step beyond freshly purified primary leukocytes, which undergo rapid apoptosis (80) and has been providing valuable insights into the development and activation of various immune cell subsets including B cells (81), macrophages (82), thrombocytes (83), neutrophilic (84) and eosinophilic granulocytes (85).

Quantitative real-time PCR (qPCR) offers another alternative to investigate particular immune cell subsets. Applied to fish, even if our repertoire of fish-specific antibodies was greatly expanded, the potential of qPCR to multiplex and to detect expression of a large number of genes in one assay will still match that of antibody-based techniques such as mass cytometry. Instead of antibodies, species-specific oligonucleotide primer sequences are derived from signature genes of red blood cells/erythrocytes (mainly spectrin- and hemoglobin-encoding genes), B cells (mainly immunoglobulin-encoding genes), T cells (mainly genes coding for components of the T-cell receptor complex), dendritic cells (e.g., *CD83*, *LAMP3*, *CD80*/*86*), thrombocytes (e.g., *ITGA2B/CD41*, *LY6G6F*), natural killer-like cells (e.g., *NCAM1*/*CD56*, *B3GAT1*, *PRF1*, *NCCRP1*), monocytes/macrophages (*CSF1R*, *MPEG1*, *ARG2*, *NOS2*, *CD40*, *CD68*, *CD74* etc.) and neutrophilic, basophilic or eosinophilic granulocytes (*CSF3R*, *MPO* or *ENPP3*). Moore and his colleagues used the Fluidigm BioMark microfluidics platform to record, for instance, the transcriptional profiles of erythroid, myeloid, lymphoid, stem and progenitor cells from zebrafish using a panel of more than 30 oligonucleotide primers (86). In mutant zebrafish, the authors discovered a novel *NKL4* ^+^ cell type, which might represent a putative cytotoxic T- or NK-like cell. A subsequent in-depth study on T cell-immunodeficient zebrafish characterized T-cell dysfunction associated with the loss of the *ZAP70* gene encoding a T-cell receptor-associated kinase (87). A multiplex qPCR study on maraena whitefish (*Coregonus maraena*) used a similar comprehensive set of oligonucleotide primers to record the cellular heterogeneity and the antigenic response of head-kidney cells from a farmed fish (88). A shortcoming of species-specific qPCR approaches is that primers are pre-selected and restricted to features that have already been identified.

This brings us to the current state of mammalian immunology which has addressed some of the problems and shortcomings faced by fish immunologists. In the past decades, large consortia such as the “Immunological Genome Project” (ImmGen) (89) and immunology working groups all over the world have compiled an impressive amount of information on mammalian immunology combining the immune phenotyping of leukocytes with the functional and molecular analysis of the key lymphocyte subsets (90, 91). Their efforts have provided critical tools for identifying various lymphocyte populations, and dissecting their developmental and activation statuses, and their effector functions (92). The deep understanding of distinct lymphocyte functions is largely based on a well-defined and comprehensive set of markers. This knowledge has been validated and further expanded in the last years by using high-throughput sequencing techniques (93), which allow for profiling of gene expression in individual subpopulations and even at the level of individual cells. Single-cell RNA sequencing (scRNA-seq) is one step beyond analysis of bulk cell populations and their whole transcriptomes, as it identifies the entirety of transcriptional changes at the level of individual cells ― independent from the presence of unique surface receptors. Overall, in-depth analyses have revealed an unexpected heterogeneity and plasticity of leukocyte populations, broadened our understanding of the development of the immune response, and even changed long-standing paradigms of leukocyte biology in health and disease (94).

Presently, in humans, the transcriptomes of individual interdigitating cell populations are providing substantial datasets and being applied towards answering the latest emerging research questions. One scRNA-seq approach, for instance, impressively documented that sex and age widely affect the susceptibility of the human immune system (95). The distinctive power and versatile applicability of the scRNA-seq technology was particularly evident in light of the COVID-19 pandemic through the rapid establishment of atlases of the peripheral immune response against SARS-CoV-2 (96, 97) and defining the mechanistic basis of the COVID-19 pathology (98). More directly relatable to the current state of fish immunology are earlier scRNA-seq studies on leukocytes from the lymphoid tissues of mammalian models such as mice. Among CD11c^+^ cells, the authors identified clusters of B cells and NK cells, among myeloid-lineage monocytes, macrophages and dendritic cells with and without LPS stimulation (99). It is by resolving the cellular heterogeneity in teleost lymphoid organs such as the spleen or kidneys that fish immunologists can begin aspiring to establishing cell atlases like those for mammals.

## Technological Advances and Its Translation to Fish Immunology

Nowadays, state-of-the-art technologies are enabling discoveries that were impossible in past decades. In life sciences, it is thanks to the combination of multi-disciplinary technologies (including physics, chemistry and computer science) that we now have practical solutions to answer fundamental questions. Among these technologies, sequencing technologies have gone through several iterations and generations: from first (Sanger method) to second and third generations with no need to amplify the reads (100), enabling new insights at the molecular level for the fields of genetics, transcriptomics and epigenetics. Concurrently, partitioning of bulk/whole specimens into their individual constituents is becoming increasingly common due to advantages such as sensitivity, accuracy, and the dilution/exclusion of contaminants as demonstrated by methods such as digital droplet PCR and fluorescence-activated cell sorting (FACS). Together, these technologies paved the way for scRNA-seq, which allows for profiling of the transcriptomes of single cells originating from heterogeneous mixtures.

Since the first publication of a single-cell transcriptome a dozen years ago (101), scRNA-seq technology has rapidly evolved and found its way into a variety of research fields. Precision, accuracy and sensitivity are the pillars of an optimal scRNA-seq protocol, which includes separating cells, reverse transcription of the individually labeled single-cell-derived RNA, an optional amplification step, preparation of a library and the subsequent sequencing, including bioinformatic analysis (102) (**Figure 1**). Nonetheless, all scRNA-seq technologies give reliable results about the expression pattern at the cellular level.

**Figure 3:**
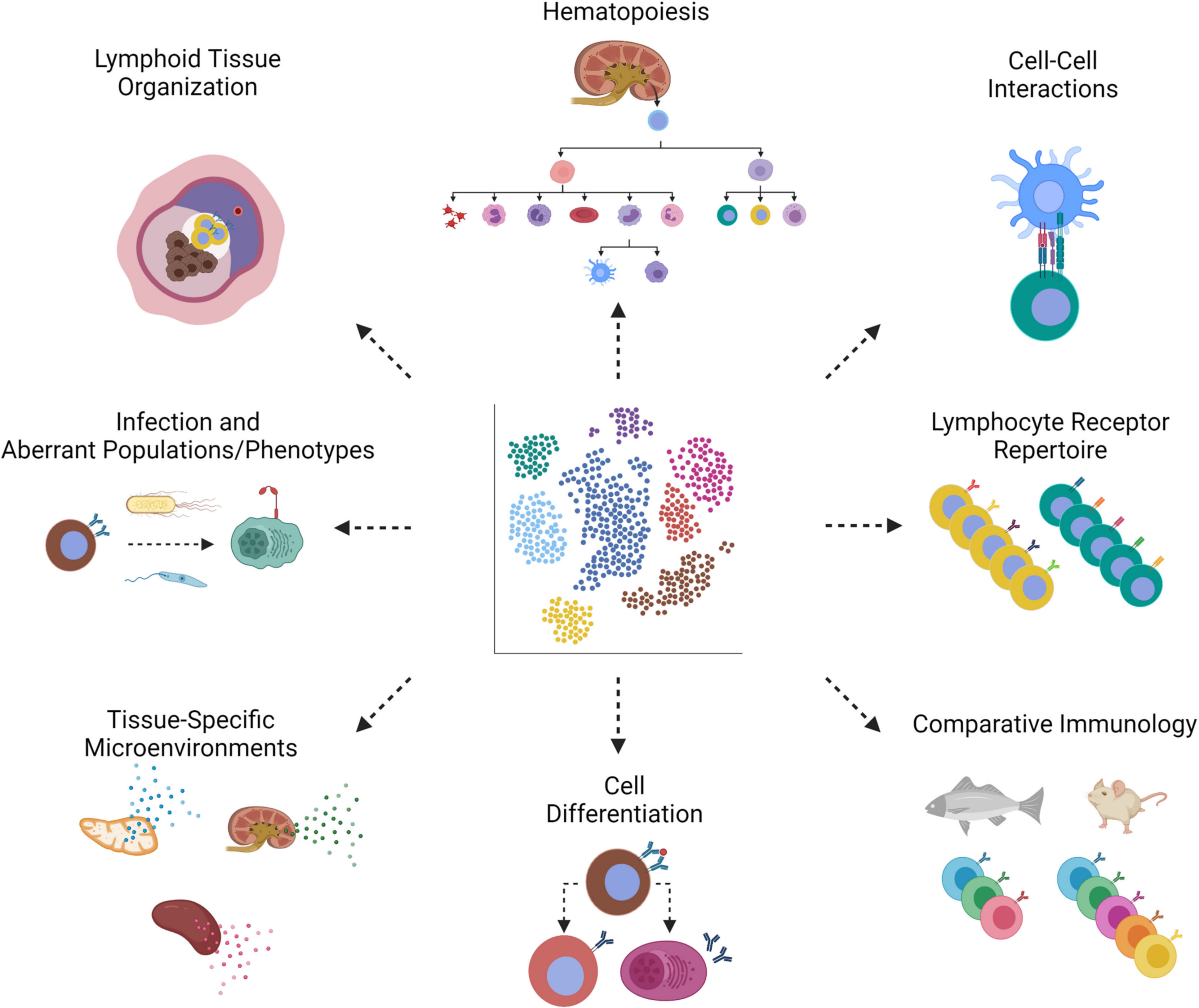
scRNA-seq applications in teleost immunology. Through scRNA-seq of teleost fishes, we can learn about the following aspects of bony fish immunology. (Clockwise, starting with the top left image, Lymphoid Tissue Organization) Depicted is splenic lymphoid tissue from fish, where we expect to discover markers of activation. The same markers can be used in techniques such as *in situ* hybridization to determine lymphoid tissue organization. Spatial transcriptomics provides both single-cell transcriptomic and spatial information in one method (108). (Hematopoiesis) The gain and loss of markers by hematopoietic stem cells throughout hematopoiesis help us piece together the developmental origins of fish leukocytes. (Cell-Cell Interactions) Complementary receptor-ligand pairs will reveal cells that interact during an immune response. (Lymphocyte Receptor Repertoire) Antigen (B or T cell) receptor repertoire sequencing benefits from single-cell technologies in which sequences of heavy and light chains remain associated with a particular clone. (Comparative Immunology) Comparison of fish and mammals can, for example, identify gene orthologues or lack thereof. (Cell Differentiation) Depicted is the differentiation of an activated B cell into a memory B or antibody-secreting cell, each distinguishable by distinct markers. (Tissue-Specific Microenvironments) The cytokine profile, for example, can be distinguished between lymphoid tissues of thymus, kidney and spleen, in order to tell us about which cell types are resident to, homing to, or retained in these tissues. (Infection and Aberrant Populations/Phenotypes) Infection of fish hosts with bacteria or parasites may give rise to populations that are rare or non-existent in homeostasis, as identified by induced or loss of markers. Created with BioRender.com.

Due to a large number of platforms and protocols that are well established, we will only briefly outline the most important systems. For in-depth looks into scRNA-seq technology beyond the scope of this review, we refer the reader to excellent publications that describe and compare the various scRNA-seq techniques in much greater detail (103–105). Undoubtedly, the Chromium system from 10× Genomics currently dominates the market. The droplet-based cell separation *via* Chromium is relatively inexpensive and less time-consuming, owing to the addition of a molecular identifier unique to cDNA of individual cells, allowing downstream applications to be performed in parallel with the highest sensitivity to ensure excellent reproducibility of the results (104). However, a relatively high (over 20 000) number of cells are required as input material. The Fluidigm C1 instrument offers an alternative, as it captures cells in up to 96 wells of a microfluidic chip. Subsequently, these separated cells can be inspected under the microscope to exclude undesired cells and enhance the eventual sequencing depth and transcript coverage (106). However, Fluidigm-based techniques are limited by cartridge sizes and thus cell size. A similar microtiter plate-based method is the SMART-seq2, which takes advantage of FACS technology for single-cell sorting, sequences full-length transcripts to generate the most mappable reads, is not restricted by cell size, and is extremely sensitive for detecting rare transcripts (105, 107). In addition to the aforementioned articles (103–105), these platforms and more are summarized in two other outstanding review articles with additional recommendations and applications for immunologists (summarized in **Figure 3**) (109, 110), and a case study on dendritic cells (110), which presents an overview of bioinformatics analyses of data on different platforms.

scRNA-seq technology heavily depends on informatics tool packages to pre-process and analyze the data. The main packages for pre-processed scRNA-seq data analysis are Seurat (111) and Scanpy (112). Both packages use accurate and reliable algorithms ensuring the reproducibility of the results. Notably, the use of deep learning, machine learning and artificial intelligence is increasingly enabling higher accuracy and scalability for large datasets. In summary, there has never been a better time to apply towards fishes, all the options, platforms, and bioinformatics tools/resources tried and tested in mammalian studies.

The scRNA-seq method is still relatively young, especially for teleost immunology. We refer the reader to **Table 2** for a summary of individual transcriptomes of fish immune cells, the main markers reported, the main populations described, and the platforms used. Although not comprehensive, we hope these studies inspire the reader to explore the possibilities offered by the technology in order to form and answer their own research questions. Existing studies have all identified previously unnoticed markers and provided astonishing new insights into the diversity of teleost immune cell populations and the evolution of vertebrate immunity. Such a comparative approach on the conservation of immune gene expression patterns generated the first single-cell transcriptomes (SCTs) of various relevant cells from zebrafish (114, 115, 119). Zebrafish is undisputedly one of the most popular teleostean models for studying cellular immune mechanisms (124, 125). Most of the teleost SCTs published to date were derived from zebrafish and are of high value for comparative immunology. Some studies are of remarkable complexity and cover the spectrum of hematopoietic cells (including various populations of innate and adaptive immune cells) in diverse tissue types of both embryonic and adult zebrafish (126, 127). More specific scRNA-seq approaches focused on zebrafish B cells that express the macrophage-characteristic marker gene *MPEG1.1* (117) or zebrafish macrophages associated with the central nervous system (CNS) that promote neurogenesis (128) and share conserved expression profiles with their counterparts in mammals (129). Meanwhile, the transcriptomes of CNS-associated macrophages from food fish species have been published as well (130). Hernández, Levraud, Cvejic and colleagues centered their research on subpopulations of innate lymphoid cells present in the gut of zebrafish (120). In addition to the comparatively abundant NK-like cells, the authors identified *NITR* ^-^ *RORC* ^+^ and *NITR* ^+^ *RORC* ^+^ populations with distinct cytokine-expression profiles, which probably correspond to mammalian innate lymphoid subpopulations involved in particular immune activation mechanisms.

**Table 2:** A summary of select literature on scRNA-seq of teleost fish immune cells.

	LEUKOCYTE POPULATION	MARKER(S)	PLATFORM	REFERENCE
*Astyanax mexicanus*	Myeloid cells	*SPI1b* and *MPX*	10×Genomics Chromium System	(113)
	Neutrophils	*CEBP1* and *MMP9*		
	Monocytes	*CSF3R* and *CD74A*		
	T cells	*CD3E*, *TCR*, and *CD4*		
	B cells	*IGKC* and *CD74A*		
*Danio renio*	T cells	*CD4, CD8A, CTLA4*, and *CD28* *BCL11B* and *TCF7* *IL10RB, CCR7, CCR9*, and *CXCR4*	SMART-seq2	(114)
	NK cells	*NITR*, *DICP*, *IL2Rb*, *S1PR5A*, *SYK, GZMB*, *PRF1*, *FAS*, and *NKL*		
	Myeloid cells	*SPI1B*, *CSF2RB*, *FCER1GL*, *HCK*, and *CFB (ZGC:158446)*		
	Hematopoietic stem cells (HSCs)	*ZFPM1*, *MEIS1B*, *GATA1A*, and *ZEB2a*	SMART-seq2	(115)
	Neutrophils	*LYZ*, *MPX*, and *CPA5*		
	B cells	*CD37*, *IGLC1S1*, and *CD79A/B*		
	B cells	*MPEG1.1, CD79A*, *CD37*, *IGHM*, and *PAX5*	InDrops RNAseq (116)	(117)
	Macrophages	*MPEG1.1, MARCO,* and *MFAP4*		
	Macrophages	*LY96*, *TLR4BB*, *TLR4AL*, and *TLR4BA*	10×Genomics Chromium System	(118)
	Monocytes	*CD74A/B*, *CTSS2.2*, and *MHC2DAB*	SMART-seq2	(119)
	Neutrophils	*CXCR4B*, *RAC2*, and *WASB*		
	HSCs	*FBL* and *PES* (pescadillo homologue)		
	Erythroid cells	*BA1*, *BA1L*, and *HBAA126*		
	Thrombocytes	*CD41*		
	Innate lymphoid cells	*NITR, RORC*, *NITR6B*, and *NITR5* (unstimulated) *IL22* and *TNF* (*V. anguillarum* stimulation) *IL13* and *GATA3* (*A. simplex* stimulation) *RORC, MCL1B*, *SOX13*, and *TNFB (*all stimulation conditions)	10×Genomics Chromium System	(120)
*Gadus morhua*	B cells	*CD22*, and *CD79*	Drop-seq (121)	(122)
	Cytotoxic T cells	*CD3, TCR, GZMB*, and *CD8*		
	Erythrocytes	*HBB*		
	Thrombocytes	*THBS1, GP1BA*, and *MPL*		
	Neutrophils	*LCE*, *EPO*, *CCL4*, *NSF1*, and *NSF2*		
	Macrophages	*CSF1R, MRC1, CSF2RA*, *CFP*, and *CCL20*		
	Plasma cells	*IGKC, CD79, IRF4*, and *SEC61*		
*Oncorhynchus mykiss*	MHC II+ B cell subpopulations	(i) *IGM, IGD, CCR9*, and *B2M* *(ii) IGT* (iii) *CD83, CCL4*, and *TNFRSF9* (iv) long non-coding RNA (general) *CXCR4*, *CD79*, *TLR12*, and *TLR13*	10×Genomics Chromium System	(27)
*Oreochromis niloticus*	B cells	*CD22*, *PIGR*, *CMRF35*, *BCL11A*, *PAX5*, and *IGLC1*	10×Genomics Chromium System	(123)
	T cells	*CD3E*, *IL2RB*, *CD7*, *CD96*, *CCR7*, *ZAP70*, *OX40*, *CXCL10*, *TOX*, and *EOMES*		
	Monocytes/macrophages	*CD209, MMP1*, *CLEC4M, C1QTNF3, MFHAS1, PLAC8*, and *PTX3*		

Almost two years after the first SCTs generated from zebrafish, Niu and colleagues elucidated the diverse subpopulations of non-specific cytotoxic cells (NCCs) (123) that correspond to mammalian natural killer (NK) cells (131). To this end, head kidney leukocytes of five Nile tilapia (*Oreochromis niloticus*) were stimulated with poly(I:C) and sorted using the 10× Genomics Chromium platform. Subsequently, the authors identified six clusters of NCCs, next to clusters of four (*CD3E^+^*) T cell populations, four (*CD209^+^ MMP1^+^*) monocyte/macrophage-populations, and three *CD22^+^* B cell populations. Remarkably, the marker *NCCRP1* (non-specific cytotoxic cell receptor protein 1) was traditionally considered as specific for all NCCs in teleosts (132), but it was here detectable in multiple clusters including monocyte/macrophage populations. The authors suggested a couple of alternative markers (*CD209-like*, *CLEC4D*, *CXCR2*, *CXCR2-like*, *HL*, *LTB4R*, *SIGLEC7*, and *STBD1*) that might be better suited for identifying NCCs in future. In addition, other markers were suggested to subdivide the NCCs from tilapia into (i) *IFIT^+^ VTCN1^+^ ARI8^+^* pre-NCCs, (ii) immature *MMP^+^ GZMK^+^ CD59^+^*, (iii) self-regulating mature *GZMB* ^+^ and (iv) memory-like *FCGR3^+^ NCAM1^+^* NCCs. The identification of NCC subpopulations in teleost fish is currently challenging with any other method in a comparably comprehensive, reliable and efficient way.

A similar approach integrating the 10× Genomics Chromium platform and a NextSeq Illumina instrument was used by the Tafalla group to dissect the composition of different immune cell populations from fish. For years, the group has been focusing its research on understanding teleost B cell functionality (72, 73). In a recent study, the rainbow trout *MHC II* ^+^ lymphocytes identified by the authors corresponded mainly to different circulating B cell subpopulations with characteristic transcriptional profiles, which reflect different stages of maturation and/or activation (27). The ten identified clusters included subpopulations of (i) *CCR9* ^+^ and *B2M* ^+^ mature B cells expressing *IgM* and *IgD*, (ii) *IgT* ^+^ B cells without *IgM* or *IgD*, (iii) *CD83* ^+^ *CCL4* ^+^ and *TNFRSF9* ^+^ activated B cells, and (iv) B cells enriched in particular long non-coding RNA (lncRNA) species, which play a crucial role during B cell activation/differentiation in naïve B cell subpopulations. Remarkably, B cells from trout lacked typical mammalian markers such as *CD19*, *CD23* or *CD24*, although other conserved signatures like genes encoding Ig heavy or light chains or isoforms of *CD79* and *CXCR4* were well detectable. Moreover, Perdiguero, Tafalla and colleagues identified novel potential markers for salmonid B cells (e.g., toll-like receptor genes 12 and 13) that have not been reported for their human counterparts yet. These findings and this approach raise the question of whether or not we would also identify trout T cells in states of activation or otherwise using *MHC II* as a pre-selected marker. In humans, *MHC II* is a marker of cytotoxic T cell activation, of diseases such as breast cancer, and potentially of good prognosis (133, 134).

The approach of fishing B cells out of a pool of immune cells based on *MHC II* expression would fail in Atlantic cod (*Gadus morhua*). Gadiform species lack the genes encoding MHC II and, additionally, the entire CD4^+^ T-cell compartment of adaptive immunity (135). MHC II molecules are important to present pathogenic antigens and activate CD4^+^ T-helper cells. Gusland and colleagues focused on the research question of how cod are still able to activate adaptive immunity and develop immunological memory (122). Their scRNA-seq analyses were performed on pre-sorted spleen and blood cell suspensions from two Atlantic cod using a Dolomite droplet generator and a subsequent sequencing on the NextSeq500 platform. They obtained 13 cell clusters containing six dominant populations of (i) (*CD22^+^ CD79^+^*) B cells, (ii) (*CD3^+^ TCR^+^ GZMB^+^ CD8^+^*) cytotoxic T cells, (iii) (*HBB^+^*) erythrocytes, (iv) (*THBS1^+^ GP1BA^+^ MPL^+^*) thrombocytes, (v) (*LCE^+^ EPO^+^ NSF^+^*) neutrophilic granulocytes, and (vi) (*CSF1R^+^ MRC1^+^ CSF2RA^+^*) macrophages. Furthermore, the authors reported clusters of splenic stromal cells (*CAVIN1^+^ FABP^+^ APOE^+^*), (*CLEVER1^+^ PLVAP^+^*) endothelial cells, transcriptionally very active plasma cells (*IRF4^+^ SEC61^+^*) among *CD79^+^* and *IG^+^* cells, (*CEACAM6^+^ KLRB1^+^*) NK-like cells, most likely (*AIF1^+^ ZNF366^+^ FLT3^+^*) dendritic cells, and cytotoxic *GATA3^+^ GZM^+^* cells. The latter *GATA3^+^ GZM^+^* cell population might represent an underexplored helper cell lineage with potential importance for lymphocyte development and adaptive responses. The clusters annotated as dendritic cells and macrophages were characterized by high expression levels of genes required for the presentation of pathogenic particles suggesting that these populations may indeed act as APCs in Atlantic cod, despite the lack of MHC II molecules. By publishing their SCT data of cod immune cells, Shuo-Wang, Guslund and colleagues paved the way to answering basic questions about the unique immune system of Gadiformes.

Castro et al. generated single-cell transcriptomes from rainbow trout B cells in a study on a “public” anti-viral hemorrhagic septicemia virus antibody clonotype (136). Although transcriptomes were mainly applied towards matching and identifying Ig light and heavy chains of the clonotype, and the same result was achieved by PCR of the single-cell cDNA, their efforts nonetheless demonstrate the potential of scRNA-seq to resolve fish lymphocytes by clonotype, the potential to clone fish antibodies for functional studies, as regularly performed in humans (137, 138), and the potential to profile the transcriptomes of individual lymphocyte clones in teleost repertoires.

The group of Peuß and Rohner used a SCT approach combining the 10× Genomics Chromium platform with Illumina HiSeq-2500 sequencing to examine the evolutionary consequences of a low parasite load for the immune system (113). They compared the transcriptomes of individual head kidney cells of one surface *versus* one cave morphotype of Mexican tetra (*Astyanax mexicanus*), because these morphotypes suffer from either a high or a low parasite abundance, respectively. scRNA-seq data indicated a significant reduction in (*SPI1b^+^ mpx* ^+^) myeloid cells, (*CEBP1^+^ LYZ^+^ PTPRC^+^*) granulo- and mono-cytopoietic cells, (*CEBP1^+^ MMP9^+^*) mature neutrophilic granulocytes and (*CSF3R^+^ CD74A^+^*) monocytes in the cave morphotype compared to surface fish. Although both morphotypes had similar abundances of B cells, the number of (*CD3E^+^ TCR ^+^CD4^+^*) T cells were increased in cavefish. The authors concluded that the immune defense of the cave morphotype relies to a greater extent on adaptive T cell responses than surface fish and hypothesized underlying genetic differences. In a subsequent scRNA-seq experiment, both morphotypes had been previously injected with lipopolysaccharide (LPS) or phosphate-buffered saline (PBS). Again, the number of T cells were increased in both groups of cavefish. In addition, more *IFNG* ^+^ T cells in LPS-injected surface fish, and the unique emergence of a particular *FOXO1B^+^ RAG^+^* B cell population in LPS-treated cavefish clearly reflected the different patterns of adaptive immune responses in both morphotypes. Although the original question was rather specific, this SCT approach provided basic insights into the composition of immune cell populations of characid fishes depending on their habitat and health status. Without such innovative approaches, the immunology of non-mammals would be set back decades and prevent the discovery of the substantial heterogeneity among immune cell populations including the varying degrees of differentiation, which also reflect the cellular activation status, location and age of fish.

Although bias is introduced, scRNA-seq can be adapted to filter cells of interest or focus on a specific gene (product) in a complex physiological context. In articles reviewed above, Perdiguero based their scRNA-seq on FACS-sorted MHC II^+^ cells (27) while Tang et al. achieved something similar using transgenic zebrafish – fish cells expressing GFP under the control of a pre-selected promoter of interest (such as *LCK* for developing or mature T cells) can be FACS-sorted, or *RAG* knockout fish can be used to eliminate T- and B-lineage lymphocyte populations (115). In another example, for almost two decades, fish immunologists have been investigating the teleost counterpart of the toll-like receptor 4 (TLR4)-dependent pathway (139, 140), which senses LPS in mammals. The unanimous scientific opinion was that LPS detection is signaled *via* alternative pathways, since essential components of the TLR4 pathway are absent in the genomes of the investigated model fish species (141, 142). A revisit of SCTs from zebrafish revealed that the components in question are expressed by a small subset of macrophages, albeit with low levels of homology to the mammalian orthologs (118). The authors demonstrated that TLR4 and its accessory molecule from zebrafish may form a functional, but less sensitive complex that recognizes LPS and activates the transcription of inflammatory genes. The characterization of the *PAX9* (paired box 9) gene is another example of an approach applying SCTs to obtain in-depth insights into the function of a particular gene (143), in this case the importance of PAX9 for granulopoiesis in zebrafish.

## scRNA-seq Applications and Future Perspectives

To understand the crosstalk and the individual contributions of leukocyte populations to the overall immune responses, to identify so far unseen relationships and to formulate novel hypotheses tailored for bony fishes, the isolation and dissection of immune cells *via* methods such as droplet encapsulation and subsequent transcriptomic analysis represents a promising approach—without pre-selected markers, bias, and the need for numerous specific antibodies. In the future, the application of scRNA-seq will enable the visualization, identification, and discovery of distinct cellular populations involved in immune responses along with their different proportions and roles. It will also allow the analysis and discovery of new cellular clusters with their respective intracellular or extracellular gene markers. In combination with tools for ontology analysis, scRNA-seq will allow the functional annotation of different cell populations highlighting the enriched biological processes and functions in the different cell clusters.

Investigating the immune system on the level of individual cells provides a broader and more integrated view of how individual parts work together to produce a particular response, without the limitations imposed by the lack of tools. Mapping the dynamic landscape of cellular identities in major lymphoid tissues in health and disease with a focus on identifying phenotypic markers of key leukocyte subsets and novel leukocyte population is only the first step. Many potential avenues of research are opened up by scRNA-seq technology, to investigate aspects of immunology that are well established in mammalian models but little explored in fish (**Figure 3**). Immune responses represent a fluid process of interaction between specialized cells, often influenced by the antagonism of pathogens. Studies investigating the dynamic changes in leukocyte signatures upon activation are poised to reveal an additional layer of complexity exemplified by cell-cell interactions during differentiation processes. Here, the bioinformatic packages such as cellChat (144), Nichenet (145) and cell2cell will be all the more valuable in identifying complementary interacting partners including the cytokines and their receptors as well as other leukocyte markers for activation, polarization, homing and trafficking. In this regard, it is worth mentioning that teleost immunity is characterized by the presence of multi-copy immune effectors including cytokines, surface receptors, and immune regulators due to teleost-specific and additional carp- or salmonid-specific rounds of whole-genome duplication events (146–148). These paralogues/ohnologues likely expand the repertoire of immune functions and represent an additional degree of specialization compared to mammals. Also for this reason, a high-quality assembly of well annotated genomes is required to adequately analyze (single-cell) RNA-seq datasets. Scientists worldwide have been working on ambitious projects such as “The Genome 10K project” (including fish genomes) (149), Fish-T1K (“Transcriptomes of 1,000 Fishes”) (150) or Fish10K (“The 10,000 Fish Genome Project”) (151) to lay the foundation for the automatic annotation of RNA-seq datasets and facilitate comparison between different species.

The analysis of the multiple interacting partners will give insight into the cellular communication between individual cells with different ligand-receptor combinations that drive differentiation towards effector subsets. Importantly, bioinformatics tools such as Scirpy (152) analyze antigen receptor repertoires coupled with trajectory inference analysis, which enables tracing of how cell clusters evolve over time, helping to elucidate the dynamism of individual lymphocyte populations over the course of an immune response. Moreover, new technologies associated with scRNA-seq such as spatial transcriptomics (108), can be of great importance allowing the spatial localization and correlation of the different identified populations *in situ* creating a link between anatomy-cell dispersion and physical cell interactions (such as cell-parasite or cell-extracellular matrix interactions). Of particular relevance for fish immunology, this technology can help study spatial organization of fish lymphoid organs during an immune response. Lastly, this technology can be readily adapted to study the immune system’s interaction with the variety of microorganisms, facilitating a more integrated view of the host-pathogen interaction and developing novel strategies for prevention and treatment. Following mammalian examples, the generated data sets could be compiled in a singular platform to facilitate the direct comparison of marker gene expression between different subsets, tissues or even species, initiating the generation of the first atlas of leukocytes in fish. Here also lies the most significant advantage of fish immunology over mammalian models. While the majority of available data stems from work on mouse or human models, the breadth of studied fish species and integration of the data in the tree of life will undoubtedly offer new perspectives on the forces driving the evolution of how adaptive responses are mounted in vertebrates.

SCTs will make studying the teleost immune system more accessible by defining key markers, garnering interest in developing reagents to study these markers and subpopulations, all the while encouraging more immunologists to explore non-conventional models from which we have much to learn. Naturally, despite its immense potential for discoveries and breakthroughs, the field cannot rely on scRNA-seq technology alone and should complement its potential with conventional immunological methods to verify the newly identified markers. Hopefully, this will reignite efforts to generate tools allowing the study of selected cell populations *via* newly available methods such as PrimeFlow, allowing the visualization of targeted RNA, or through revisiting the old-fashioned methods of monoclonal antibody production. Only through achieving this cascade of milestones can we hope to understand the inner workings of fish immune systems and revolutionize the field of fish immunology.

## Author Contributions

TK and AR conceptualized the idea for the review. JTHC designed and created all figures. All authors performed the literature search, analyzed cited references, and wrote parts of the review article. All authors contributed to the article and approved the submitted version.

## Funding

This work was funded by the Ministry of Education, Youth and Sports-Inter-action, USA of the Czech Republic (MŠMT- LTAUSA) - LTAUSA19108. This work was also funded by the MoMV project, financed by the European Union and the state of Mecklenburg-Vorpommern - 7302-MV-II.12-LM-001.

## Conflict of Interest

The authors declare that the research was conducted in the absence of any commercial or financial relationships that could be construed as a potential conflict of interest.

## Publisher’s Note

All claims expressed in this article are solely those of the authors and do not necessarily represent those of their affiliated organizations, or those of the publisher, the editors and the reviewers. Any product that may be evaluated in this article, or claim that may be made by its manufacturer, is not guaranteed or endorsed by the publisher.
